# Characterisation of the French ferret population, husbandry, reported medical care and feeding habits

**DOI:** 10.1017/jns.2017.51

**Published:** 2018-01-31

**Authors:** Géraldine Blanchard, Maud Marsot, Roselyne Bourassin, Bernard-Marie Paragon, Jean-Jacques Benet, Adeline Linsart

**Affiliations:** 1 Animal Nutrition Expertise SARL, Antony, France; 2 University Paris Est, ANSES, Laboratory for Animal Health, Epidemiology Unit, Maisons-Alfort, France; 3 Ecole Nationale Vétérinaire d'Alfort, Maisons Alfort, France; 4 CHV St Martin Bellevue, St Martin Bellevue, France

**Keywords:** Ferrets, Husbandry, Feeding habits, Reported diseases

## Abstract

Ferrets have become a common companion animal. But no data are available on the French population of ferrets. The goal of the survey was to characterise this population, including demographic characteristics, husbandry, reported medical care and feeding habits. Complete data were available for 1205 pet ferrets in 709 households. Most ferrets (86·1 %) lived indoors, 1 % received only artificial lighting, and 47 % chewed their cage. For 60 % of the ferrets, body weight was higher in winter and lower in summer. Neutered ferrets (58·5 % of males and 62·9 % of females) appeared lighter than intact ferrets of comparable age. A majority (52·4 %) of ferrets received a mix of commercial foods and fresh foods, but 28·6 % were offered no commercial foods. Data were analysed using several multivariable logistic regression models including age, sex, castration, food type and artificial lighting developed for four clinical outcome (lethargy and/or insulinoma, dental problems, diarrhoea and/or bird-seed stools and alopecia). Predictors of four clinical outcomes (lethargy, dental disease, diarrhoea and alopecia) were examined using multivariable logistic regression, with age, sex, neuter status, food type and artificial lighting as the exposure variables. Aged ferrets were more likely to have lethargy, insulinoma, dental problems and alopecia. Ferrets with artificial lighting were more likely to show alopecia. Additionally, ferrets fed commercial food only or a mixed diet (both commercial food and fresh food) were more likely to have lethargy, insulinoma, dental problems, diarrhoea and/or bird-seed stools compared with ferrets fed fresh food only. We also found a significant association between neutering and alopecia. It is to our knowledge the first description of the French population of the ferret as a companion animal.

Ferrets (*Mustela putorius furo*) are strict carnivorous mustelids related to mink. They were historically used for hunting, but have become an increasingly popular pet in Europe and in North America. Some diseases are common in pet ferrets, including insulinoma, hyperadrenocorticism and lymphoma^(^^1^^)^ in North America but few data are available about the pet ferret population in France. Nutritionists and wildlife and exotic animal specialists are often questioned about the optimal diet for ferrets, and the relationship between diet and disease^(^^2^^–^^5^^)^. Some recommendations have been offered using the cat as a model^(^^6^^)^, but some breeders have pointed out the deficiencies of this model, and have recommended fresh meat-based foods with very low carbohydrate content. New foods for ferrets are also available commercially^(^^7^^)^. There have been many hypotheses about links between food, neutering, husbandry and various diseases, but there are few published data, and more reviews than original studies so far.

The goal of the present study was first to characterise the diet, husbandry and health of the French ferret population, and to assess the effect of diet and husbandry on dental health, digestive disease, insulinoma, fatigue and lethargy, adrenal disease and alopecia.

## Materials and methods

### Survey

The survey was designed as a descriptive survey with a quantitative objective. The target population was all pet ferrets in France. The source population was French-speaking owners of ferrets with Internet access. The recruitment of ferret owners was advertised through newspapers and online specialised forums. Owners answered on a voluntary and anonymous basis.

### Procedure

A preliminary version of the survey was tested for understanding and duration with owners of ferrets that were not thereafter included in the survey. A validated questionnaire (Supplementary material) of ninety-one closed questions was developed and available online between June and October 2012. Each survey required 20–30 min to be completed.

The first part of the questionnaire was dedicated to individual data regarding each ferret; the second part included more general questions about the household to identify owners’ habits and applied to all ferrets living in the same household. The demographic characteristics of the sample were studied together with husbandry, reported medical care and feeding habits.

### Statistics

#### Descriptive analysis

Data analysis was run via epidemiology software (Epi Info 3; Centers for Disease Control and Prevention) and a statistical package (BiostaTGV). Descriptive data were reported as actual counts and the percentage of respondents. Differences between responses were determined by use of the *χ*^2^ test and *t* test in the studied population. Differences were considered significant at values of *P* < 0·05.

Associations between neuter status, sex, husbandry, food composition and distribution, mode of neutering and reported medical conditions were tested using univariate analysis. Then data were pooled as follows for the quantitative analysis for each outcome: lethargy, weakness, decreased activity, stand up only for food and declared insulinoma were pooled as lethargy ± insulinoma; diarrhoea and green or bird-seed stools pooled as diarrhoea ± bird-seed stools; dental problems including broken or impaired and injured teeth and teeth extraction pooled as dental problems; permanent tail alopecia, troncular alopecia and hair loss, and ‘ferrets naked except on the head, adrenal disease, adrenal tumour’ and ferrets declared with adrenal disease pooled as alopecia ± adrenal disease.

#### Quantitative analysis

Statistical computations were performed in R-3.2.0 (R Core Team). Four multivariable logistic regression models were developed using the status for (i) insulinoma and/or lethargy, (ii) diarrhoea and/or bird-seed stools, (iii) dental problems (fracture, extraction or tartar) and (iv) adrenal disease of each ferret as the outcome measure and the ferret as the unit of analysis. Six variables and two interactions were considered: age (three classes less than 1 year old, 1 to 3 years old, over 3 years old), sex (two classes), castration (yes or no), use of daylight (natural or artificial with or without natural light), commercial (fresh only *v.* commercial only).

The model was built following two steps:

- The variables considered were tested for collinearity, including multi-co-linearity analysis to ensure a mean variance inflation factor of <10 before being used in the multivariable models^(^^8^^)^.
- A multivariable model was then constructed with a generalised linear model^(^^9^^)^ using a binomial distribution (logit link). Model assessment was based on Akaike's information criterion. Results were expressed as OR and 95 % CI. Some interactions were tested but not significant and not retained in the final model (i.e. commercial × sex, commercial × age, diet × sex, diet × age).

A second run of models was done including the variable DIET (commercial food only *v.* mixed food *v.* fresh food only) instead of the variable PETFOOD (commercial food *v.* no commercial food at all). This second analysis was done in order to test the specific effect on symptoms of ferrets of the use of a mix of commercial and fresh food.

## Results

### Population

Data were available for 1205 ferrets, located in eighty-five of the ninety-five French departments. Out of the studied population (Table 1), 52·8 % were male, and 60·6 % were neutered (58·5 % of males were castrated, 62·9 % of females were spayed). Neutering was performed by surgery (79·4 %) or by hormonal implants with deslorelin. Sterilisation by implant increased with age, representing 30·8 % of ferrets older than 2 years. Neutering occurred significantly (*P* < 0·05) earlier in females, with 55·8 % of females but only 36·7 % of males neutered before 9 months old.

**Table 1.** Distribution of sex, age and body weight (BW) in the population studied (*n* 1205) (Numbers of animals and percentages; mean values and standard deviations)

			BW of sexually intact (g)*			BW of neutered/spayed (g)*		
	*n*	%	Mean	sd	*n*	Mean	sd	*n*
Male	636	52·8			201			287
<6 months	72	11·3	976	295	38	600	–	1
6–12 months	48	7·5	1495	573	23	1242	289	12
1–3 years	305	48·0	1428	326	104	1325	317	132
3–5 years	138	21·7	1328	274	28	1164	267	94
>5 years	73	11·5	1305	787	8	1127	231	48
Female	569	47·2			157			289
<6 months	63	11·1	630	233	35	*–*	*–*	0
6–12 months	54	9·5	805	168	22	892	336	23
1–3 years	261	45·9	855	324	79	738	174	135
3–5 years	118	20·7	827	160	16	738	138	80
>5 years	73	12·8	846	238	5	697	233	51

* Data unknown for 148 males and 123 females.

Body weight of neutered ferrets was significantly (*P* < 0·05) lower than that of entire ferrets of comparable age and sex (Table 1). Ferrets neutered by surgery were significantly lighter than those sterilised by implant, whatever the age (*P* < 0·05).

Body weight was reported to vary seasonally for 60 % of ferrets, being significantly higher in winter and lower in summer (*P* < 0·05).

### Origin and lifestyle

Ferrets had been obtained most commonly from other pet ferret owners (*n* 701/1051; 59·3 %) and less often from ferret breeders (16·1 %), pet shops (14·7) or associations (14·7 %).

Most ferrets lived exclusively or mainly indoors (86·1 %); 6·6 % lived outdoors only. About 59·5 % of ferrets had access to more than 10 m^2^ of cage surface area twice per d. Owners reported that 47 % of ferrets chewed their cage. This behaviour did not vary with the origin of the animal or with the sex, but significantly increased (*P* < 0·05) as available living surface decreased, and decreased with age (*P* < 0·05).

In 77·2 % of the 723 respondent households, the ferret was not the only companion animal: 65·8 % owned more than one ferret, 63·3 % cats, 61·9 % dogs, 22·5 % rodents, 9·5 % birds, 9·2 % rabbits, 8·5 % reptiles, and 13 % other animals.

In 64 % of the households, combined natural and artificial lighting was reported in the ferret's living room. Lighting was natural only in 35·0 %, while it was artificial only in 1·0 %.

During winter, 33·9 % of ferrets were exposed to artificial light in the evening and 33·1 % in the morning and evening.

### Feeding habits

Most ferrets (60·7 %) were fed by one person in the household.

Among the 1051 ferrets, 71·4 % received commercial food, including kibble (99·1 %) or wet food (17·1 %), on a regular basis. Indeed, 91·8 % of them received dry food (kibble) daily, while wet food was delivered daily for 26·6 % of the ferrets, one to six times per week for 16·4 %, once per week for 16·4 % and less than once per week for 43 %.

Ferrets had *ad libitum* access to kibble in 85·1 % of cases, while wet food was delivered mainly once (30 %) or twice (41 %) daily, and treats mostly (75·7 %) once daily.

The commercial foods provided were formulated for ferrets (50 % of the kibbles, 34 % of the wet), for cats (49 % of the kibbles, 54 % of the wet), or for other species.

Among the 1051 ferrets, 51·9 % (*n* 546) have access to both pet food, either dry or wet or both, and fresh ingredients, and 28·6 % (*n* 301) received no commercial food at all, but a variety of fresh ingredients: 79·8 % meat and offal (*n* 604, with 53·6 % at least once per week and 21·4 % daily), 53·9 % whole prey (*n* 408, with 31·8 % daily), empty carcases (*n* 99, with 24 % at least once per week), 25·9 % plant and fish oils (*n* 273, with 44 % at least once per week), 20·9 % raw vegetables and fruits (*n* 147, with 24·5 % at least once per week but only 0·9 % daily), and 13·1 % sources of complex carbohydrates (rice, potato, pasta, etc.) (*n* 91, with *n* 28 at least once per week).

These ingredients were delivered raw for 100 % of the whole prey (98·3 % of owners keep them in the freezer, 30 % in the refrigerator), 93·3 % of the empty carcases, 93·1 % of the meats and offal, 99·4 % for the oils, and 88·7 % for vegetables and fruits.

Among the 467 household that reported keeping foods frozen, the defrosting method used was mostly the refrigerator for 79·7 %, at room temperature for 31·9 %, in the micro-waves oven for 12·8 %, in a bain-marie for 6·9 %.

Among the 1051 ferrets, treats were reportedly offered once or several times per week in 64·8 % (*n* 681) of ferrets.

A change in feeding habits was reported in 40·6 % of ferrets (*n* 1003). In 18·6 % (*n* 397), the main reason for the change was a health issue cited in the following decreasing prevalence order: digestive troubles (*n* 28), kibble intolerance (*n* 3), insulinoma treatment/prevention and lethargy (*n* 9), underweight (*n* 8), overweight (*n* 2), lymphoma (*n* 4), hyporexia (*n* 3), urolithiasis (*n* 3), renal failure (*n* 2), renal tumour (*n* 1), Ca deficiency (*n* 1), and others (*n* 10).

The removal of some ingredients was reported to efficiently limit the observed troubles in the 1003 ferrets: kibble (65 %), whole prey (14·7 %), meats and offal (10·8 %), commercial supplements and treats (11·1 %), commercial wet food (7·8 %), dairy products (7·2 %), vegetables and fruits (5·9 %), human cookies (5·9 %), and convalescence support foods (4·2 %).

### Clinical features

Most ferrets (1156/1205) had already consulted a veterinarian, mostly for vaccination (*n* 975), identification (*n* 581), a disease (*n* 560), travelling information (*n* 138), reproduction issues (*n* 108), and for geriatric consultation (*n* 96).

For each of the 1205 ferrets, the owner was asked if the ferret has already suffered from any disease/trouble so far, as an open question. The most common answers were diarrhoea and green or bird-seed stools (32·5 % of ferrets) and dental problems including broken or impaired and injured teeth (21·2 %), respiratory troubles such as dyspnoea, coughing and snuffle (14·9 %), unexplained weight loss not attributable to seasonal variation (13·3 %), lethargy, weakness, decreased activity, stand up only for food (11 %), tartar (11 %), locomotion troubles, osteoarthritis, hind limb weakness, lameness (5·5 %), hair loss, alopecia except on the head, adrenal disease, adrenal tumour (4·2 %), urinary or urination troubles, cystitis, crystals or blood in urine (3·6 %), dental extraction (3·1 %), eyes with white or blue coloration, cataract (2·9 %), abnormal glycaemia, pancreatitis, insulinoma, diabetes (2·6 %), kidney disease (1·4 %), and heart disease (0·8 %).

A specific question was asked to distinguish physiological seasonal alopecia (67·6 % of our ferrets), truncal alopecia (on the back and the sides of the body) (0·5 %), permanent tail alopecia (2·6 %), transitory tail alopecia (9 %), and pruritis (1·5 %).

At the time of the survey, 105 ferrets (10·1 %) were reported to be medically treated for: digestive disease (*n* 31), insulinoma or hypoglycaemia (*n* 11), cancer (*n* 9 including *n* 5 lymphoma), otitis or ears trouble (*n* 9), adrenal disease and alopecia (*n* 9), respiratory troubles (*n* 7), gingivitis (*n* 2), corneal ulcer (*n* 2), stroke (*n* 2), digestive parasitism (*n* 2) and eight other conditions (*n* 1 each).

History of diarrhoea after consumption of identified foods was reported in 45 % (*n* 503/*n* 1079) of ferrets: eggs (*n* 116), kibble (*n* 73), dairy products (*n* 69), various meats (*n* 42), day-old chicks (*n* 36), fruits (*n* 34), vegetables (*n* 23), bone and raw food (barf) diet (*n* 21), liver (*n* 20), treats for ferrets (*n* 19), canned cat food (*n* 17), chocolate (*n* 10), offal (*n* 10), and (fewer than *n* 10 each) cream, butter, prescription diets for cats, cat milk, candies, biscuits, vitamin supplements, dry fruits, convalescence diets for humans, ice cream, oil, and eighteen other foods.

Insulinoma and/or lethargy were more likely with ageing, and less likely in ferrets having no pet food at all; diarrhoea and/or bird-seed stools were more likely in neutered ferrets, and less likely in ferrets having no pet food at all; dental problems were more likely with ageing and less likely with females (Table 2). Alopecia and/or adrenal disease were more likely for ferrets over 3 years old, for females, or for ferrets receiving artificial lighting in winter (compared with ferrets receiving natural light only at this time of the year).

**Table 2.** Odds ratios and their associated 95 % confidence intervals and effective *n* obtained from the final models (PETFOOD with the variable commercial, DIET with the variable DIET) on ferrets

	Insulinoma ± lethargy			Diarrhoea ± bird-seed stools			Dental problems†			Alopecia ± adrenal disease		
Variable	*n*	OR	95 % CI	*n*	OR	95 % CI	*n*	OR	95 % CI	*n*	OR	95 % CI
Model PETFOOD												
Commercial												
No	291	Reference		289	Reference		288	NR		195	NR	
Yes	691	3·0*	1·4, 7·5	714	2·0*	1·5, 2·8	694	NR		508	NR	
Age												
<1 year	170	Reference		172	Reference		169	Reference		111	Reference	
1–3 years	479	2·8*	1·2, 8·3	483	0·9	0·6, 1·4	478	4·3*	2·1, 9·8	349	4·0	0·8, 74·2
>3 years	333	9·2*	4·0, 26·6	348	1·6	1·0, 2·4	335	15·1*	7·4, 35·1	243	23·8*	5·0, 428·0
Sex												
Male	508	Reference		525	Reference		511	Reference		373	Reference	
Female	474	1·8	0·7, 5·0	478	0·8	0·6, 1·0	471	0·5*	0·4, 0·8	330	2·1*	1·1, 4·0
Castration												
No	383	NR		390	Reference		385	Reference		278	NR	
Yes	599	NR		613	1·4*	1·1, 2·0	597	1·4 1·0, 2·0		425	NR	
Daylight												
Natural	–	NT		–	NT		–	NT		161	Reference	
Artificial ± natural	–	NT		–	NT		–	NT		542	6·5*	2·3, 27·3
Model DIET												
Diet												
Fresh	291	Reference		289	Reference		288	Reference		195	NR	
Mixed	569	3·0*	1·4, 7·5	587	2·1*	1·5, 2·9	569	1·4	·0, 2·1	421	NR	
Commercial	122	2·1	0·8, 6·2	127	1·8*	1·1, 2·8	125	0·7	0·4, 1·2	87	NR	
Age												
<1 year	170	Reference		172	Reference		169	Reference		111	Reference	
1–3 years	479	2·5	1·0, 7·5	483	0·9	0·6, 1·4	478	4·3*	2·1, 9·8	349	4·0	0·8, 74·2
>3 years	333	7·6*	3·2, 22·7	348	1·6	1·0, 2·4	335	15·1*	7·4, 35·3	243	23·8*	5·0, 428·0
Sex												
Male	508	Reference		525	Reference		511	Reference		373	Reference	
Female	474	1·8	0·7, 4·9	478	0·8	0·6, 1·0	471	0·5*	0·4, 0·7	330	2·1*	1·1, 4·0
Castration												
No	383	Reference		390	Reference		385	Reference		278	NR	
Yes	599	1·4	0·9, 2·4	613	1·5*	1·1, 2·0	597	1·3	0·9, 2·0	425	NR	
Daylight												
Natural	–	NT		–	NT		–	NT		161	Reference	
Artificial ± natural	–	NT		–	NT		–	NT		542	6·5*	2·3, 27·3

NR, not retained in the final model; NT, not tested in the model.

* Significant variables retained (the value 1·0 not included in the 95 % CI of the OR).

† Dental problems included fracture, extraction or tartar.

## Discussion

Among the information collected, some were expected, such as the large proportion of households owning more than one ferret (65·8 %), the sexual dimorphism and higher body weight for males, the seasonal change in body weight with heavier ferrets before winter, the large proportion of ferrets biting their cage at least on a regular basis (47 %), the large proportion of neutered ferrets (60·6 %), the change of method of neutering, still mainly by surgery (79·4 %) but with an increase of the use of implants.

The body weight of neutered ferrets was significantly (*P* < 0·05) lower than entire ferrets of comparable age and sex, and this unexpected variation could not be related to diet or age.

The high level of medical care of the ferret population studied, with 91 % having already been examined by a veterinarian and 76·7 % of ferrets vaccinated, may be a bias, as owners answering such a survey may be the most committed to the health of their pet ferret.

Among all reasons for consulting a veterinarian, digestive troubles were the most represented, with 32·5 % of ferrets already affected, and dental problems were second with 21·2 %, then 11 % had already shown shown lethargy and 2·6 % suffered of hypoglycaemia. The high prevalence of these symptoms and/or diseases and the hypothesis of their relation to environmental origin, such as diet and lighting, justified the interest of the multivariate analysis, with all the limits of such a survey.

### Lethargy and/or insulinoma

Ferrets eating commercial food are three times more likely to develop lethargy and/or insulinoma and hypoglycaemia. Insulinoma is known as a very common disease, with a prevalence of 20–25 % of middle-aged and old ferrets (range 2–7 years old)^(^^10^^)^. Even if lethargy may be a general sign of disease and not only of insulinoma^(^^1^^,^^3^^,^^10^^–^^13^^)^, lethargy and hind limb weakness have been identified in 91–95 % of cases of insulinoma in two studies^(^^13^^,^^14^^)^. The differential diagnosis of lethargy and hypoglycaemia in ferrets must include insulinoma, severe hepatic disease, paraneoplastic effects, sepsis, malnutrition, starvation^(^^3^^,^^10^^)^ and other less common diseases such as cardiopathy and abdominal pain. However, most of these signs are quite obvious and lead to medical advice. Decreased activity and a reluctance to move are generally not considered as abnormal by the owner but more a sign of ageing, especially at the early stage of insulinoma^(^^1^^,^^13^^)^.

The risk factors of insulinoma have not been clearly identified so far^(^^3^^,^^10^^)^. Carbohydrate-rich pet foods have been suspected to induce hyperplasia of pancreatic *β*-cells. Some authors have suggested a genetic origin (MEN-like, multiple endocrine neoplasia) considering the high frequency of several endocrine tumours in the same individual^(^^3^^,^^11^^)^. We could not confirm any link between signs of insulinoma and of adrenal disease in our study.

### Diarrhoea

Diarrhoea, green stools and bird-seed stools are common for ferrets. The latter are considered as a sign of maldigestion by veterinarians for exotic species, but no origin has been clearly identified so far. They appear twice as common in ferrets consuming commercial food, or mixed food compared with ferrets having no pet food at all.

Among all ferrets having commercial food, 99 % of them had at least some dry food. The link between the consumption of commercial pet food, and lethargy and insulinoma and diarrhoea may be the carbohydrate concentration of commercial food. The consumption of starch is not included in the natural diet of ferrets. But their reaction to the consumption of starch may be linked to different mechanisms:

- If starch and sugars are digested, and glucose absorbed, then glucose can enter into circulation. Absorption is usually very efficient in animal species physiologically not adapted to deal with high amounts of glucose^(^^15^^)^. This stimulates insulin secretion more than low-carbohydrate diets, and could exhaust the *β*-cells after a few years of consumption of such a regimen, and lead to lethargy and insulinoma.
- If starch and sugars are poorly digested due to a lack of appropriate enzymes, then undigested starch can reach the gut and be fermented, which may lead to bacterial overgrowth, loose stools and a decreased digestibility due to an acceleration of transit, which could explain bird-seed stools, and diarrhoea. The microbiome may also be affected by this diet and mechanism.

The link between the consumption of eggs and diarrhoea, as described by some ferret owners, may have an explanation in the composition of the egg white^(^^16^^)^, which contains ovomucoid with trypsin-inhibitory activity^(^^17^^)^, which lowers protein digestibility of raw egg protein.

### Dental problems

From this study, female ferrets seemed protected from dental problems compared with males. There are no data available and no clear physiological explanation, but there may be a behavioural explanation. In the authors’ experience, ferret owners have a closer relationship with males than with female ferrets. Females are considered more active and inquisitive, and more at risk of biting especially after 1 year of age, which does not encourage close observation of teeth by the owner. Oral examination of ferrets often required (49/63 ferrets) general anaesthesia^(^^18^^)^, and dental troubles in females might be underestimated.

The association between dental problems and captivity has been described in captive black-footed ferrets (*Mustela nigripes*)^(^^19^^)^. This article suggested a link to the food, but with no evidence. We could not link the dental problems or even the teeth fractures to a behaviour of biting the cage. We could link the dental problems to different variables in the one-way analysis – tartar in ferrets over 3 years old consuming dry food daily compared with occasionally or never, and fractures in ferrets having commercial food compared with fresh food only – but it could not be confirmed in the multivariate model. There are several hypotheses: (i) there is no link between diet and dental disease in ferrets; (ii) the number of cases in each category of dental problem was too small and pooling all dental problems diluted potential associations; (iii) the origin of teeth fractures may be different from those of tartar; (iv) dental problems are underestimated by ferret owners, as they are quite difficult to identify.

### Alopecia and adrenal disease

Suggested risk factors of adrenal disease are neutering, prolonged photoperiod and genetic.

We could not identify any effect of neutering. Older ferrets appeared much more likely to show such signs. A significant association between artificial lighting in winter and alopecia and/or adrenal disease confirmed a previous hypothesis^(^^3^^,^^5^^)^. Adrenal disease is described in both males and females, and a higher risk for female ferrets in this study, but this may be related to a misinterpretation of the signs. Indeed, the differential diagnosis of adrenal disease especially in the females includes prolonged oestrus and ovarian remnant, which both induce a prolonged hyperoestrogenism, inducing a bilateral and symmetrical alopecia, as well as a vulvar oedema^(^^10^^,^^11^^,^^20^^,^^21^^)^. In male ferrets, a testicular tumour must be included in the differential diagnosis^(^^22^^)^.

The two models studied (PETFOOD and DIET) led to similar results, which suggests that a mixed feeding of commercial and fresh food did not lower the risks associated with a regimen based on commercial food only. Dry food was not distinguished separately in our models, as 99·1 % of ferrets having commercial food were receiving at least some dry food. Cat food and kitten food have been and are still extensively recommended for ferrets, but kitten wet food found in the European market may contain between 10 and 14 % energy from carbohydrates, kitten dry cat food 16 to 45 %, canned adult cat food 2 to 33 % and dry adult cat food 16 to 45 % (survey of eighty-four cat foods in France all available at veterinary practices; brands available in supermarkets are not included). Dry food for ferrets all contains carbohydrate, due to the manufacturing extrusion process which requires starch to produce a kibble.

So far, to lower carbohydrate intake, it is possible to recommend either a balanced homemade diet (containing meat or fish, rapeseed oil, fish oil, wheat bran, and an adapted mineral–vitamin supplement) formulated by a specialist in veterinary animal nutrition, or a balanced commercial wet food for kittens or ferrets with the lowest possible carbohydrate concentration (less than 0·5; which requires a calculation from the label information: 100 – % moisture – % crude protein – % crude fat – % fibre – % ash).

Even if some biases can be pointed out, first, by the selection of passionate ferret owners who answered the questionnaire and, second, by the lack of medical evidence of the reported health conditions, and results must be confirmed, it is to our knowledge the first description of the French population of pet ferrets. Also, the present study enrolled a large number of ferrets, making a good base for further investigations to validate (or not) the suggested links pointed out here between food and various conditions.

### Conclusion

More studies are required to confirm the links suggested by this study, between feeding habits, husbandry and diseases, as case–control studies, using populations of ferrets followed by veterinarians to validate the diagnosis.

So far, we can recommend to ferret owners to respect the strict carnivore character of pet ferrets, to avoid artificial lighting, to bring male and female ferrets to the veterinarian for a routine check-up, including dental examination on a regular basis, and to provide very low-carbohydrate foods.

Additionally, veterinarians could endorse the education of ferret owners, teaching that a pet ferret is still a ferret, an obligate carnivore, with specific requirements and providing precise and safe husbandry, nutrition and feeding recommendations.

## Supplementary material

For supplementary material accompanying this paper visit https://doi.org/10.1017/jns.2017.51 click here to view supplementary material
