# Supplementary material for: Characterisation of the French ferret population, husbandry, reported medical care and feeding habits
Source: J Nutr Sci. 2018 Jan 31;7:e4. doi: 10.1017/jns.2017.51 (PMC5793136; doi:10.1017/jns.2017.51)
Supplement: Supplementary file 1 [file S2048679017000519sup001.pdf]

ANNEXE 1 : Questionnaire

***1. Quel est l'âge de votre furet?**

- ◯ moins de six mois
- ◯ entre 6 mois et 1 an
- ◯ entre 1 et 3 ans
- ◯ entre 3 et 5 ans
- ◯ plus de 5 ans

***2. Votre furet est:**

- ◯ un mâle
- ◯ une femelle

***3. Votre furet est il stérilisé?**

- ◯ oui
- ◯ non

***4. Par quelle méthode votre furet a-t-il/elle été stérilisée(e)?**

- ◯ chirurgicale
- ◯ implant(durée d'action entre 1,5 et 2 ans)
- ◯ injection répétée 1 à 2 fois par an

***5. Cet implant a-t-il déjà été renouvelé?**

- ◯ oui
- ◯ non
- ◯ ne sait pas

***6. A quel âge a eu lieu la stérilisation?**

- ◯ entre 3 et 6 mois
- ◯ entre 6 et 9 mois
- ◯ entre 9 et 12 mois
- ◯ entre 1 et 2 ans
- ◯ à plus de 2 ans
- ◯ ne sait pas

***7. D'où vient votre furet?**

- ◯ de chez un éleveur
- ◯ d'une animalerie
- ◯ de chez un particulier (voisin, ami, éleveur amateur)
- ◯ d'une association

***8. Est ce votre premier furet?**

- ◯ oui
- ◯ non

9. Votre furet mord les barreaux de sa cage:

- ◯ jamais
- ◯ de temps en temps
- ◯ tous les jours

10. Votre furet va à l'exterieur (en heures par semaine):

	jamais	moins d'1 h/semaine	1 à 3h/semaine	3 à 7h/semaine	plus de 7h/semaine
balade en laisse	◯	◯	◯	◯	◯
pelouse	◯	◯	◯	◯	◯
aire de jeux dédiée ou parc fermé	◯	◯	◯	◯	◯
bac à sable/terre extérieur	◯	◯	◯	◯	◯

***11. Avez vous déjà consulté un vétérinaire pour votre furet (même pour un simple avis)?**

- ◯ oui
- ◯ non

12. Au cours de la vie de votre furet, indiquez si vous avez consulté un vétérinaire pour...

	jamais	1 fois	plusieurs fois
vaccination	○	○	○
identification	○	○	○
stérilisation	○	○	○
voyage	○	○	○
maladie	○	○	○
vieillesse	○	○	○
reproduction	○	○	○
autre	○	○	○

13. Votre furet a-t-il déjà eu un des examens suivants au cours de sa vie avec vous?

	oui	non	ne sait pas
prise de sang ou bilan sanguin	○	○	○
radiographie ou échographie pour le coeur	○	○	○
radiographie ou échographie pour une autre raison que pour le coeur	○	○	○

14. Est-ce que votre furet prend un traitement actuellement?

○ oui

○ non

15. Sous quelle forme de médicaments? (plusieurs réponses possibles)

☐ comprimés

☐ sirop

☐ injections

☐ aérosol

☐ autres

16. Pour quelle raison? Pour quelle maladie?

17. Votre furet est il vacciné tous les ans?

- oui
- non
- ne sait pas

18. Contre quelle(s) maladie(s)?

- rage
- maladie de Carré
- rage et maladie de Carré
- ne sait pas

19. Le vétérinaire a-t-il déjà réalisé une recherche de coronavirus (=ECE= Entérite catarrhale épizootique) sur votre furet?

- oui
- non
- ne sait pas

20. Votre furet a-t-il des contacts avec des furets extérieurs à ceux de votre foyer?

- oui
- non

21. A quel rythme?

- rarement
- une à plusieurs fois par mois
- une à plusieurs fois par semaine

22. La robe de votre furet comporte-t-elle des tâches blanches sur la tête? (ex: marquage panda, blaze ou flamme, badger ou blaireua)

- ◯ oui
- ◯ non
- ◯ ne sait pas

23. Avez vous effectué un test auditif (PEA) ?

- ◯ oui
- ◯ non
- ◯ ne sait pas

24. Quel en est le résultat?

	Audition normale	Audition diminuée	Surdité
oreille droite	◯	◯	◯
oreille gauche	◯	◯	◯

***25. Votre furet perd-il ses poils?**

- ◯ non
- ◯ uniquement la mue saisonnière sur l'ensemble du corps
- ◯ oui, parce qu'il se gratte
- ◯ oui, un peu partout tout le temps
- ◯ oui, surtout sur le dos et sur les côtés (il est presque nu)
- ◯ oui, sur la queue mais pas tout le temps
- ◯ oui, sur la queue tout le temps (les poils ne repoussent pas)

26. Avez vous observé un jaunissement des poils de votre furet?

- ◯ oui
- ◯ non
- ◯ ne sait pas

27. Ce jaunissement a-t-il lieu?

- ◯ plutôt l'hiver
- ◯ plutôt l'été
- ◯ toute l'année

28. A votre connaissance, votre furet a-t-il une maladie du coeur?

- ◯ oui
- ◯ non
- ◯ ne sait pas

29. A votre connaissance, votre furet souffre-t-il ou a-t-il souffert d'une des maladies suivantes?

	oui	non	ne sait pas
problème pour uriner, trouble urinaire, cystite,sang dans les urines, urines rouges, blocage urinaire, calculs ou cristaux	○	○	○
furet tout nu sauf sur la tête, maladie surrénalienne, tumeur des glandes surrénales	○	○	○
blocage des reins, insuffisance rénale	○	○	○
trop ou pas assez de sucre dans le sang, pancréatite, insulinome, diabète, glycémie anormale	○	○	○
selles granuleuses, diarrhée, selles vertes	○	○	○
difficultés à se déplacer, faiblesse du train arrière, arthrose, boiterie	○	○	○
amaigrissement inexpliqué (hors variation de poids saisonnière)	○	○	○
léthargie, faiblesse, nette diminution d'activité, ne joue plus, ne se lève que pour manger	○	○	○
aspect blanc ou bleuté des yeux, cataracte	○	○	○
dents cassées ou abimées	○	○	○
extraction de dents déjà réalisée ou à réaliser	○	○	○
détartrage déjà réalisé ou tartre visible sur les dents	○	○	○
difficultés à respirer, toux, snuffle	○	○	○

***30. Vous diriez, de manière générale, que votre furet a des selles**

○ liquides comme de l'eau

○ molles à pâteuses (crème épaisse, bouse)

○ moulées mais molles (au toucher)

○ moulées dures

○ ne sait pas

***31. Vous diriez, de manière générale, que votre furet a des selles de couleur (plusieurs réponses possibles)**

- ☐ jaune
- ☐ marron
- ☐ vert
- ☐ noir
- ☐ rouge
- ☐ ne sait pas

***32. Vous diriez, de manière générale, que votre furet a des selles**

- ○ homogènes (texture lisse)
- ○ hétérogènes (lisse+ petits morceaux d'os, plumes...)
- ○ granuleuses (avec des éléments comme des petites billes)
- ○ ne sait pas

***33. Est-il déjà arrivé que la consommation d'un aliment par votre furet entraine des diarrhées (selles liquides ou très molles et/ou vertes)?**

- ○ oui
- ○ non
- ○ ne sait pas

34. De quel(s) aliment(s) s'agissait-il? (lister en séparant les aliments d'une virgule)

____

***35. Votre furet est-il?**

- ○ plus gros en hiver qu'en été
- ○ plus maigre en hiver qu'en été
- ○ pareil en hiver et en été (a 100 grammes près)
- ○ ne sait pas

36. Cette variation a-t-elle été vérifiée par une pesée?

- ◯ oui
- ◯ non
- ◯ ne sait pas

37. Poids le plus faible relevé

en grammes

38. Poids le plus fort relevé

en grammes

***39. Vous diriez de votre furet qu'il est?**

- ◯ trop maigre
- ◯ maigre
- ◯ ni gros, ni maigre, juste bien
- ◯ légèrement trop gros
- ◯ trop gros
- ◯ ne sait pas

40. Quel est le poids actuel de votre furet?

en grammes

41. Votre furet boit-il?

- ◯ oui
- ◯ non
- ◯ ne sait pas

42. Que boit-il? (plusieurs réponses possibles)

- ☐ eau
- ☐ lait
- ☐ jus de fruit
- ☐ soda

Autre (veuillez préciser)

43. Dans quel type de récipient votre furet boit-il principalement?

- ○ bol en plastique
- ○ bol en verre
- ○ bol en céramique
- ○ bol métallique
- ○ biberon
- ○ autre

***44. Votre furet mange t-il des aliments complets (indiqué sur l'étiquette) achetés tout prêts? (croquettes, boites, barquettes, sachets)**

- ○ oui
- ○ non, jamais

45. Quels aliments complets (indiqué sur l'étiquette) achetés tout prêts votre furet mange t-il? (plusieurs réponses possibles)

	pour furet/fureton	pour chat	pour chaton	pour chien/chiot	autre
croquettes	☐	☐	☐	☐	☐
boites/barquettes/sachets	☐	☐	☐	☐	☐

***46. A quelle fréquence votre furet mange t-il des aliments complets?**

	tous les jours	1 à 6 fois par semaine	1 fois par semaine	moins souvent	jamais
croquettes	○	○	○	○	○
boite/barquette/sachet	○	○	○	○	○

***47. Votre furet consomme t-il, même rarement, d'autres aliments que des aliments complets (proies, repas que vous préparez vous même, compléments, friandises...)?**

- ○ oui
- ○ non,absolument jamais

48. Pour les catégories d'aliments suivants, indiquer s'ils sont donnés crus ou cuits, et à quelle fréquence ils sont consommés par votre furet

	frequence	cuisson
proies entières	▾	▾
carcasse vidée	▾	▾
viande et abats	▾	▾
huile végétale ou huile de poisson	▾	▾
legumes et fruits	▾	▾
féculents (riz, pommes de terre, pâtes)	▾	▾

49. Pour les catégories d'aliments suivants, indiquez à quelle fréquence ils sont consommés

	tous les jours	2 à 6 fois par semaine	moins d'une fois par semaine
Friandises type gateaux, céréales du petit déjeuner, bonbon, pizza...	○	○	○
Laitages (lait, fromage, yaourt, crème, beurre)	○	○	○
Aliments complémentaires pour furets, friandises achetées, treats	○	○	○
Aliments minéraux et vitaminés (pâtes, gouttes, liquides...)	○	○	○
Aliments de convalescence (pour chat ou pour furet)	○	○	○

50. Parmi les aliments carnés ou d'origine animale suivants, indiquez ceux que vous donnez, même occasionnellement (plusieurs réponses possibles)

- ☐ viande/poisson (steak, viande, blanc de volaille, coeur, filet de poisson)
- ☐ rognon
- ☐ foie
- ☐ caille entière (avec abats et tête, sans les plumes)
- ☐ proie entière vivante
- ☐ proie entière fraichement tuée
- ☐ proie entière congelée
- ☐ "steak" de volaille broyée congelé type Dogador, Poher, Prodia
- ☐ cou ou aile de poulet ou autre volaille
- ☐ os avec cartilage

Autre (veuillez préciser)

51. Parmi les aliments suivants, indiquez ceux que vous donnez à votre furet

- ☐ beurre
- ☐ crème fraîche
- ☐ huile de colza ou de soja
- ☐ huile de pépin de raisin ou isio 4
- ☐ huile de tournesol ou de maïs
- ☐ huile d'olive
- ☐ huile de poisson
- ☐ huile de foie de morue
- ☐ huile de paraffine

52. Parmi les aliments suivants, indiquez ceux que vous donnez à votre furet

- ☐ lait (vache, chèvre, brebis)
- ☐ lait délactosé (matin léger, lait pour chat, lait sans lactose)
- ☐ lait maternisé pour chaton
- ☐ fromage
- ☐ yaourt
- ☐ fromage blanc
- ☐ crème fraîche

55. Avez vous donné ou donnez vous, même occasionnellement, des aliments pour convalescence? Si oui, lesquels?

- [ ] Carnivore Care (Oxbow)
- [ ] Fortol (MSD)
- [ ] a/d (Hill's)
- [ ] Convalescence support (Royal Canin)

Autre (veuillez préciser)

[ ]

***56. Avez-vous déjà changé le type d'alimentation (de croquettes à carné par exemple) de votre furet au cours de sa vie?**

- ( ) oui
- ( ) non

57. Quels types d'aliments donniez vous que vous ne donnez plus maintenant? (plusieurs réponses possibles)

- [ ] croquettes
- [ ] aliments complets humides
- [ ] proies entières
- [ ] carcasse vidée
- [ ] viandes et abats
- [ ] huile végétale ou huile de poisson
- [ ] légumes et fruits
- [ ] féculents (riz, pommes de terre, pâtes)
- [ ] laitages
- [ ] gâteaux
- [ ] aliments complémentaires, friandises achetées, treats
- [ ] aliments minéraux et vitaminés (pâtes, gouttes, liquides...)
- [ ] aliments de convalescence (pour chat ou furet)

58. Avez-vous changé l'alimentation de votre furet en raison d'un problème de santé?

- ( ) oui
- ( ) non

Si oui, précisez de quel problème il s'agit

[ ]

53. Parmi les friandises suivantes, indiquez celles que vous donnez à votre furet

- [ ] Ferret bits (Totally Ferret)
- [ ] Kitty Snacks
- [ ] Friandise à mâcher Dokas
- [ ] Croq brin Crackers Tyrrol
- [ ] Friandise pour furet Francodex

Autre (veuillez préciser)

54. Quels compléments de calcium, minéraux ou vitamines, donnez vous à votre furet, même occasionnellement?

- [ ] Levure de bière en paillette ou en comprimés
- [ ] Huile de foie de morue
- [ ] Ferretone
- [ ] Ferrevite
- [ ] Multivitamine Gimpet
- [ ] Ebi-vet
- [ ] Sunshine Factor
- [ ] Nutrigel plus
- [ ] Petphos Ca/P=1,3
- [ ] Petphos Ca/P=2
- [ ] Petphos pelage
- [ ] Sofcanis croissance
- [ ] Sofcanis félin
- [ ] Tonivit
- [ ] Vit'i5 Little Ca
- [ ] Vit'i5 Little Ca/P=3
- [ ] Aucun

Autre (veuillez préciser)

***59. Arrive-t-il que sa gamelle ne contienne pas d'aliment dans la journée ?**

	jamais	rarement	régulièrement	toujours entre deux repas
croquettes, friandises sèches	○	○	○	○
aliments humides	○	○	○	○

60. Combien de repas (même petits) donnez vous par jour à votre furet?

	1	2 ou 3	4 et plus	disponible à volonté
croquettes	○	○	○	○
aliments humides	○	○	○	○
friandises	○	○	○	○

61. Dans quel récipient recoit-il son repas? (plusieurs réponses possibles)

- ☐ une gamelle qui lui est réservée
- ☐ une gamelle accessible aux autres furets/animaux de la maison
- ☐ un distributeur à réservoir d'aliment sec
- ☐ un distributeur à minuterie d'aliment sec
- ☐ une assiette comme vous

***62. Votre furet a-t-il des cachettes à nourriture (dans sa cage, sa pièce de vie...)?**

- ○ oui
- ○ non
- ○ ne sait pas

***63. Est-ce le premier questionnaire de notre étude auquel vous répondez?**

- ○ oui
- ○ non, j'en ai déjà rempli un pour un autre de mes furets

64. En général, à quelle fréquence nettoyez-vous les "cachettes" à nourriture de votre/vos furet(s)? (s'il y en a)

○ 1 fois par an au moins

○ environ 1 fois par mois

○ environ 1 fois par semaine

○ environ 1 fois par jour

○ plusieurs fois par jour

65. Pesez-vous les aliments que vous donnez à votre/vos furet(s)?

○ oui, tous les jours

○ de temps en temps

○ non

66. Où distribuez principalement les repas?

○ dans la cage ou la pièce de vie dédiée au(x) furet(s)

○ à table avec vous

○ dans la maison, en dehors de la cage ou de la pièce de vie dédiée au(x) furet(s)

67. Si vous en donnez, où achetez vous principalement les aliments complets (croquettes, boîtes, barquettes, sachets)?

	le plus souvent	parfois	jamais
supermarché	○	○	○
animalerie	○	○	○
vétérinaire	○	○	○
internet	○	○	○
autre	○	○	○

68. Si vous en donnez, combien de temps au maximum ces aliments complets restent-ils disponibles dans la gamelle après distribution avant que vous ou votre(vos) furet(s) ne la vidiez totalement?

	moins d'1 heure	1 à 8 heures	8 à 12 heures	de 12 heures à plusieurs jours
croquettes	○	○	○	○
boite/barquette/sachet	○	○	○	○

69. Dans le cas où vous donnez des proies entières, où les conservez vous? (plusieurs réponses possibles)

- ☐ réfrigérateur
- ☐ congélateur

Autre (veuillez préciser)

[ ]

70. Si vous utilisez des aliments congelés pour nourrir votre (vos) furet(s), quel mode de décongélation utilisez vous? (plusieurs réponses possibles)

- ☐ à l'air libre
- ☐ au réfrigérateur
- ☐ au micro-ondes
- ☐ au bain marie
- ☐ autre

***71. Combien de personnes à la maison nourrissent votre(vos) furet(s) (hors période de vacances ou d'absence)?**

- ○ vous uniquement
- ○ 1 personne seulement (pas vous)
- ○ 2 personnes au total
- ○ 3 personnes ou plus

***72. Votre(vos) furet(s) vit(vivent):**

- ○ exclusivement à l'extérieur
- ○ principalement à l'extérieur
- ○ principalement à l'intérieur
- ○ exclusivement à l'intérieur

73. Quelle est la surface de vie disponible pour votre(vos) furet(s)?

	jusqu'à 1 m2 (cage)	de 1 à 3 m2 (tower)	de 3 à 10 m2 (tower, balcon, petite pièce dédiée)	plus de 10 m2 (chambre dédiée, accès libre dans la maison, volière extérieure)
en journée	○	○	○	○
la nuit	○	○	○	○
le matin et le soir	○	○	○	○

***74. Le mode d'éclairage de la pièce de vie principale de votre(vos) furet(s) est:**

- ◯ uniquement naturel
- ◯ naturel+ artificiel
- ◯ uniquement artificiel

***75. En hiver, votre(vos) furet(s) bénéficie-t-il(s) d'un éclairage artificiel?**

- ◯ le matin seulement
- ◯ matin et soir
- ◯ le soir seulement
- ◯ à aucun moment

76. Votre(vos) furet(s) est(sont) dans le noir complet (ni lumière du jour, ni éclairage par lumière artificielle, ni veilleuse de nuit):

	moins de 6 H par nuit	de 6 à 9 H par nuit	de 9 à 12 H par nuit	plus de 12 H par nuit
été	◯	◯	◯	◯
hiver	◯	◯	◯	◯

***77. Y a-t-il des personnes qui fument en présence de votre(vos) furet(s) (même fenêtre ouverte)?**

- ◯ jamais
- ◯ souvent (tous les jours)
- ◯ parfois (1 à 2 fois par semaine)

***78. Avez-vous d'autres animaux à la maison?**

- ◯ oui
- ◯ non

79. Lesquels? (plusieurs réponses possibles)

- ☐ furet(s)
- ☐ chat(s)
- ☐ chien(s)
- ☐ rongeur(s)
- ☐ lapin(s)
- ☐ oiseau(x)
- ☐ reptile(s)
- ☐ autre(s)

80. Combien d'autres furets possédez vous?

- ◯ 1
- ◯ 2
- ◯ 3
- ◯ plus de 3

81. A votre connaissance existe-t-il un lien de parenté entre vos furets?

- ◯ oui
- ◯ non
- ◯ ne sait pas

82. A votre connaissance, vos furets viennent-ils du même élevage?

- ◯ oui
- ◯ non
- ◯ ne sait pas

83. Y a -t-il des informations que nous ne vous avons pas demandé à propos de votre (vos) furet(s) et dont vous aimeriez nous faire part?

84. Si vous acceptez d'être contacté(e) pour une enquête ultérieure du même type à propos du furet, vous pouvez nous laisser une adresse email valide (elle ne sera pas communiqué à des tiers)

85. Etes vous?

- ◯ un homme
- ◯ une femme

86. Quel est votre âge?

- ◯ moins de 16 ans
- ◯ 16 à 20 ans
- ◯ 21 à 30 ans
- ◯ 31 à 40 ans
- ◯ plus de 40 ans

87. Etes vous?

- ○ célibataire
- ○ en couple

88. Y a-t-il des enfants (moins de 16 ans) dans votre foyer?

- ○ oui
- ○ non

89. Dans quel pays vivez vous?

[ ]

90. Où vivez vous?

Code postal [ ]

Ca y est, c'est terminé !
Si vous avez un ou plusieurs autres furets, merci de remplir un questionnaire par animal, en retournant sur la page d'accueil www.vet-nutrition.com/furet .
Les principaux résultats de cette enquête seront disponibles d'ici quelques mois, mais pour vous remercier de votre participation, si vous avez indiqué votre adresse email, vous recevrez les résultats en avant-première.
Si vous avez oublié de nous laisser votre adresse email, il n'est pas trop tard... vous pouvez le faire ci-dessous
Encore merci de nous aider à mieux connaître pour mieux soigner nos amis les furets !

91. Votre adresse email (attention à l'entrer sans faute !)

[ ]
